# N-Acetylgalactosamine-4-sulfatase (Arylsulfatase B) Regulates PD-L1 Expression in Melanoma by an HDAC3-Mediated Epigenetic Mechanism

**DOI:** 10.3390/ijms25115851

**Published:** 2024-05-28

**Authors:** Sumit Bhattacharyya, InSug O-Sullivan, Joanne K. Tobacman

**Affiliations:** Jesse Brown VAMC and Department of Medicine, University of Illinois Chicago, Chicago, IL 60612, USA; ramabha@uic.edu (S.B.); insug@uic.edu (I.O.-S.)

**Keywords:** arylsulfatase B, chondroitin sulfate, HDAC3, galectin-3, c-Jun

## Abstract

The effects of the enzyme N-acetylgalactosamine-4-sulfatase (Arylsulfatase B, ARSB), which removes the 4-sulfate group at the non-reducing end of chondroitin 4-sulfate, on the expression of PD-L1 were determined, and the underlying mechanism of PD-L1 expression was elucidated. Initial experiments in human melanoma cells (A375) showed that PD-L1 expression increased from 357 ± 31 to 796 ± 50 pg/mg protein (*p* < 10^−11^) when ARSB was silenced in A375 cells. In subcutaneous B16F10 murine melanomas, PD-L1 declined from 1227 ± 189 to 583 ± 110 pg/mg protein (*p* = 1.67 × 10^−7^), a decline of 52%, following treatment with exogenous, bioactive recombinant ARSB. This decline occurred in association with reduced tumor growth and prolongation of survival, as previously reported. The mechanism of regulation of PD-L1 expression by ARSB is attributed to ARSB-mediated alteration in chondroitin 4-sulfation, leading to changes in free galectin-3, c-Jun nuclear localization, HDAC3 expression, and effects of acetyl-H3 on the PD-L1 promoter. These findings indicate that changes in ARSB contribute to the expression of PD-L1 in melanoma and can thereby affect the immune checkpoint response. Exogenous ARSB acted on melanoma cells and normal melanocytes through the IGF2 receptor. The decline in PD-L1 expression by exogenous ARSB may contribute to the impact of ARSB on melanoma progression.

## 1. Introduction

Agents to modify checkpoint inhibition by PD-L1 (programmed death ligand 1) and its immune cell receptor (PD-1) are widely used to treat malignant melanoma and other malignancies. However, response to treatment is unpredictable and may not be sustained [1,2,3,4,5]. Treatment with exogenous, bioactive Arylsulfatase B (ARSB; N-acetylgalactosamine-4-sulfatase), the enzyme that removes 4-sulfate groups at the non-reducing end from chondroitin 4-sulfate (C4S), was recently shown to reduce tumor progression and improve the survival of C57BL/6J mice with B16F10 subcutaneous melanomas [6]. The potential impact of ARSB on PD-L1 expression was unknown and is addressed in this report.

In prior work, a decline in ARSB was associated with the increasing invasiveness of human melanoma cell lines, as well as with more aggressive human prostate and colonic cancers and with malignant mammary cells [7,8]. As ARSB activity decreased, chondroitin 4-sulfation increased, leading to increased binding of SHP2 (PTP11; non-receptor tyrosine phosphatase) and reduced binding of galectin-3 with C4S [8,9,10,11,12,13]. Subsequent effects attributed to decline in SHP2 availability include increases in phospho-ERK1/2 and phospho-p38 MAPK and transcriptional effects. These include hypermethylation of the DKK3 promoter and reduced expression of DKK (Dickkopf WNT pathway signaling inhibitor) 3, leading to activation of Wnt signaling in prostate cells [11]. Other transcriptional events followed the silencing of ARSB and increased availability of galectin-3. These include increased expression of CSPG4 and CHST15 in melanoma cells [6,7], as well as increased expression of versican in prostate epithelial cells [10], of Wnt9A in colonic epithelial cells [12], and of HIF-1α in bronchial and colonic epithelial cells [13]. The transcriptional events initiated by changes in SHP2 or galectin-3 binding with C4S, which follow either a decline or an increase in ARSB activity, exerted profound effects on vital cell processes. The relationship between these ARSB-C4S-initiated transcriptional mechanisms and immune-mediated effects on melanoma tumor proliferation has not been addressed previously.

Initial experiments showed that silencing ARSB in melanoma cells increased the expression of PD-L1 mRNA and protein and, inversely, treatment by exogenous ARSB reduced PD-L1 expression in human and mouse melanoma cells [8]. The experiments detailed in this report were performed to clarify the mechanism by which ARSB regulated PD-L1 expression in the subcutaneous B16F10 mouse melanomas, in human A375 melanoma cells, and in normal human melanocytes.

Reports in the literature have presented several different transcriptional mechanisms affecting PD-L1 expression, including specific transcription factors and epigenetic mechanisms involving methylations and histone deacetylations [14,15,16,17,18,19,20,21,22,23,24,25,26,27,28,29,30,31]. HDACs 3, 6, and 8 have been implicated in effects on histone acetylation in melanoma [14,15,21,27,28,30,31]. Changes in ARSB or chondroitin-4 sulfate have not previously been associated with the regulation of histone acetylation and HDACs. The findings that follow support the impact of ARSB and chondroitin 4-sulfation on galectin-3, c-Jun, HDAC3, and H3 on the regulation of PD-L1 expression in melanoma and suggest that ARSB may help with checkpoint inhibition.

## 2. Results

### 2.1. PD-L1 Expression Is Reduced by Exogenous ARSB and Increased by ARSB Knockdown

**Figure 1:**
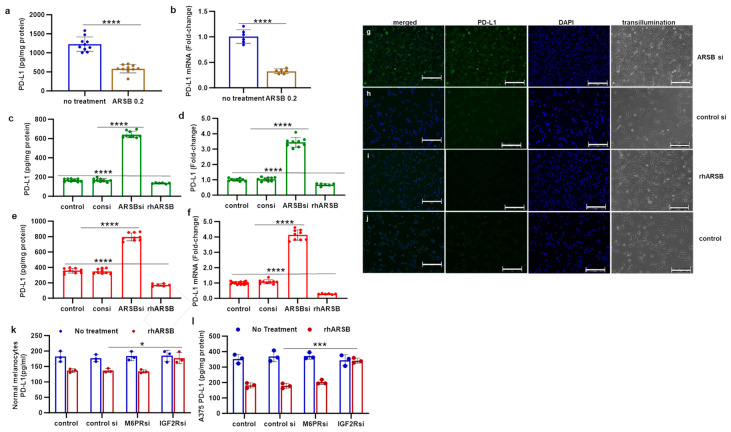
PD-L1 expression and protein in B16F10 subcutaneous melanomas, normal melanocytes, and A375 melanoma cells following ARSB silencing and exposure to exogenous, bioactive ARSB. (**a**,**b**) In the mouse melanomas, PD-L1 protein (n = 9) and mRNA expression (n = 6) are reduced following treatment with exogenous ARSB (*p* < 0.0001). (**c**,**d**) In normal human melanocytes, ARSB silencing increases the protein and mRNA expression of PD-L1 (*p* < 0.0001, n = 9). (**e**,**f**) Similarly, in the malignant A375 cells, PD-L1 protein and mRNA are increased by ARSB silencing and reduced by rhARSB (*p* < 0.0001, n = 9). The baseline PD-L1 value is greater in the malignant than in the normal cells. (**g**–**j**) Immunohistochemistry of cultured A375 cells stained for PD-L1 demonstrates marked increase following ARSB siRNA (**g**) and decrease following exogenous ARSB (**i**), compared to the control siRNA (**h**) and control (**j**). (**k**,**l**) In the normal melanocytes and the A375 cells, silencing of the mannose-6-phosphate receptor (M6PR) by siRNA did not block the effect of exogenous ARSB on PD-L1 expression. In contrast, silencing of the calcium-independent insulin-like growth factor 2 receptor (IGF2R) by siRNA almost completely blocked the rhARSB-induced decline in PD-L1. *p*-values are <0.0001, determined by an unpaired *t*-test, two-tailed, with unequal variance and n ≥ 6. [ARSB = arylsulfatase B = N-acetylgalactosamine-4-sulfatase; consi = control siRNA; IGF2R = insulin-like growth factor 2 receptor; M6PR = mannose-6-phosphate receptor; PD-L1 = programmed death ligand-1; rh = recombinant human; si = small interfering siRNA; 0.2 = 0.2 mg/kg treatment dose]. **** represents *p* ≤ 0.0001, *** represents *p* ≤ 0.001, and * is for *p* ≤ 0.05. Scale bar is 150 um.

PD-L1 was measured in tumor tissue from B16F10 subcutaneous melanomas in C57Bl/6J mice, which had been treated by local injection of exogenous, bioactive ARSB (rhARSB). Mice were treated with ARSB 0.2 mg/kg on days 2, 7, and 14 following tumor inoculation, as previously reported [6]. The mean tumor volume in the total group of ARSB-treated mice was 0.23 ± 0.15 cm^3^ (n = 28) and 1.05 ± 0.7 cm^3^ in the control group (n = 9) on day 16 (*p* = 0.006, unpaired *t*-test, two-tailed, unequal variance) [6]. The survival at 21 days post tumor inoculation was greater in the ARSB-treated mice than in the control mice (*p* = 0.039, log-rank test). PD-L1 protein and mRNA expression were measured in tumor disease obtained at necropsy. The values were lower in the ARSB-treated mice, compared to saline-treated controls (Figure 1a,b).

Measurements of PD-L1 protein and mRNA were also performed following ARSB silencing or exposure to rhARSB (1 ng/mL × 24 h) in cultured normal human melanocytes (Figure 1c,d) and in A375 human melanoma cells (Figure 1e,f). ARSB silencing increased PD-L1 expression, and exogenous ARSB reduced PD-L1 expression (*p* < 0.0001, *p* < 0.0001; unpaired *t*-test, two-tailed, unequal variance). The immunohistochemistry of A375 cells confirms marked increase in PD-L1 immunostaining following ARSB knockdown (Figure 1g), compared to control siRNA (Figure 1h). A decline in PD-L1 immunostaining is evident following treatment with rhARSB (Figure 1i), compared to the control (Figure 1j). Overexpression of ARSB by transfection in A375 cells produced a similar decrease in PD-L1 expression (Supplementary Figure S1b). Transfection reduced PD-L1 mRNA expression to 28% (n = 6) of the baseline, which is comparable to a decline to 31% (n = 6) of the baseline by rhARSB (Supplementary Figure S1b).

The reduction in PD-L1 expression following exogenous ARSB in the normal melanocytes (Figure 1k) and the A375 cells (Figure 1l) was inhibited by the silencing of the insulin-like growth factor 2 receptor (IGF2R), but not by silencing the mannose-6-phosphate receptor (M6PR) [32].

### 2.2. Exogenous ARSB Reduces Free Galectin-3 Due to Increased Galectin-3 Binding with Chondroitin 4-Sulfate

**Figure 2:**
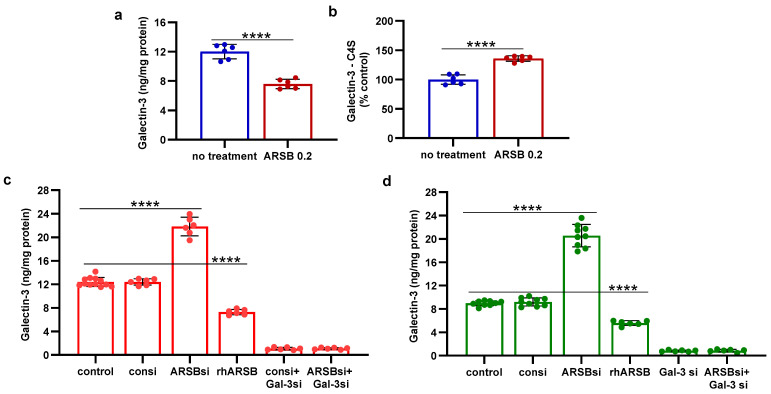
Inverse effects of silencing ARSB and rhARSB on galectin-3 due to altered binding with chondroitin 4-sulfate. (**a**) In the mouse melanoma tissue, treatment by recombinant ARSB reduced the free galectin-3, as measured by ELISA (*p* < 0.0001, n = 6). (**b**) The decline in free galectin-3 is attributed to the increased binding of galectin-3 with chondroitin 4-sulfate, which was detected following immunoprecipitation of a tumor tissue with a C4S antibody. (**c**) In the A375 melanoma cells, free galectin-3 increased when ARSB was silenced and declined following exogenous ARSB (*p* < 0.0001), consistent with altered galectin-3-C4S binding following a decline or an increase in ARSB activity. Galectin-3 siRNA increased free galectin-3 protein. (**d**) Similarly, in the normal human melanocytes, ARSB siRNA increased free galectin-3, and exogenous ARSB reduced free galectin-3 (*p* < 0.0001). *p*-values are < 0.0001, determined by unpaired *t*-test, two-tailed, with unequal variance and n ≥ 6 for all determinations. [ARSB = arylsulfatase B = N-acetylgalactosamine-4-sulfatase; consi = control siRNA; gal-3 = galectin-3; rh = recombinant human; si = small interfering siRNA; 0.2 = 0.2 mg/kg treatment dose]. **** represents *p* ≤ 0.0001.

The mechanism by which PD-L1 expression was regulated by ARSB was evaluated based on evidence from previous experiments [8,10,12,13]. Changes in free galectin-3 occurred due to ARSB-induced alteration of chondroitin 4-sulfate (C4S)-galectin-3 binding [6,10,12,13,33]. Increased C4S-galectin-3 binding occurred when ARSB activity was increased and C4S was less sulfated, leading to reduced free galectin-3. Inversely, reduced C4S-galectin-3 binding occurred when ARSB activity was reduced, leading to increased free galectin-3. In the melanoma tissue of the C57BL/6J mice, treatment with exogenous ARSB reduced the free galectin-3 (*p* < 0.0001) (Figure 2a). The immunoprecipitation of galectin-3 with C4S showed an increase in the binding of galectin-3 with C4S following ARSB (*p* < 0.0001) (Figure 2b). Experiments in A375 cells (Figure 2c) and in normal human melanocytes (M0) (Figure 2d) indicate increases in free galectin-3 when ARSB was silenced (*p* < 0.0001), and reductions following exposure to exogenous galectin-3 (*p* < 0.0001).

In contrast to the observed binding of galectin-3 with chondroitin 4-sulfate, the quantity of PD-L1 that co-immunoprecipitated with chondroitin sulfate (CS) in the A375 cells was negligible (0.10 pg/mg CS), with no significant difference following ARSB siRNA or exogenous ARSB (Supplementary Figure S1a).

### 2.3. Impact of Galectin-3 on Phospho-(Thr183/Tyr185)-JNK and Nuclear c-Jun

**Figure 3:**
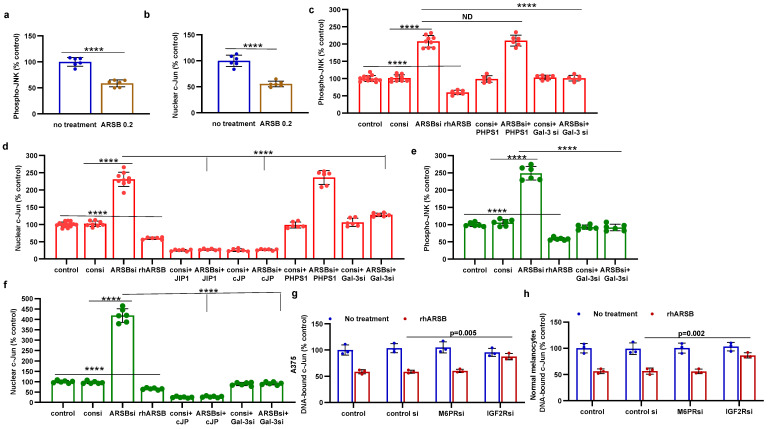
Inverse effects of ARSB siRNA and rhARSB on phospho-JNK and nuclear c-Jun. (**a**,**b**) In the mouse melanoma tissue, phospho-(Thr183/Tyr185)-JNK and nuclear c-Jun declined following exogenous ARSB (*p* < 0.0001, n = 6). (**c**,**d**) In the A375 cells, ARSB siRNA increased phospho-JNK (*p* < 0.0001, n = 9) and nuclear c-Jun (*p* < 0.0001, n = 9). In contrast, rhARSB reduced both phospho-JNK (*p* < 0.0001, n = 6) and c-Jun (*p* < 0.0001, n = 6). The SHP-2 inhibitor PHPS1 did not reduce the effect of ARSB siRNA. In contrast, galectin-3 siRNA significantly reduced the ARSB si-induced increases. JIP1, a JNK-selective inhibitory peptide, and cJP, a c-Jun mimetic peptide, both completely blocked the increases caused by ARSB siRNA. (**e**,**f**) Similarly, in the normal melanocytes, phospho-JNK and DNA-bound c-Jun were affected by ARSB siRNA and recombinant ARSB (*p* < 0.0001, n = 6). Galectin-3 silencing blocked the effect of ARSB siRNA. (**g**,**h**) In the A375 cells and the normal melanocytes, the effect of exogenous ARSB on DNA-bound c-Jun was inhibited by the IGF2R siRNA (*p* = 0.005, *p* = 0.002; n = 3, n = 3), but not by M6PR silencing. *p*-values were determined by unpaired *t*-test, two-tailed, with unequal variance. [ARSB = arylsulfatase B = N-acetylgalactosamine-4-sulfatase; consi = control siRNA; gal-3 = galectin-3; IGF2R = insulin-like growth factor 2 receptor; JIP1 = JNK-selective inhibitory peptide; cJP = c-Jun mimetic peptide; JNK = c-Jun N-terminal kinase 1; M6PR = mannose-6-phosphate receptor; ND = no significant difference; PD-L1 = programmed death ligand-1; PHPS1 = SHP2 inhibitor; rh = recombinant human; si = small interfering siRNA; 0.2 = 0.2 mg/kg treatment dose]. **** represents *p* ≤ 0.0001.

Galectin-3 enhanced the activation of AP-1 in prior experiments [10,12,13], so the impact of exogenous ARSB-induced decline in galectin-3 on DNA-bound c-Jun and on c-Jun N-terminal kinase 1 (JNK) was addressed. In the B16F10 melanoma tumor tissue and in the normal melanocyte (M0) and malignant A375 cells, the effects of exogenous ARSB and of ARSB and galectin-3 knockdown were evaluated. In the B16F10 tumors, both phospho-(Thr185/Tyr185)-JNK and nuclear c-Jun decreased following treatment with exogenous ARSB (Figure 3a,b) (*p* < 0.0001, *p* < 0.0001). Phospho-JNK declined by ~41% and nuclear c-Jun by ~45%. In the A375 (Figure 3c,d) and normal melanocyte cell lines (Figure 3e,f), exogenous ARSB reduced phospho-JNK and c-Jun (*p* < 0.0001, *p* < 0.0001). Galectin-3 silencing blocked the ARSB siRNA-induced increases in phospho-JNK and c-Jun. The c-Jun mimetic peptide cJP and the c-Jun inhibitory peptide JIP1 inhibited ARSB siRNA-induced increases in DNA-bound c-Jun. PHPS1, a small-molecule chemical inhibitor of the SHP2 (PTPN11), did not inhibit the ARSB siRNA-induced increase in phospho-JNK or DNA-bound c-Jun in the M0 and A375 cells. The IGF2R siRNA almost completely reversed the rhARSB-induced decline in DNA-bound c-Jun in the A375 (Figure 3g) and M0 (Figure 3h) cells.

### 2.4. HDAC3 Activity and Expression Is Modulated by ARSB and Galectin-3

**Figure 4:**
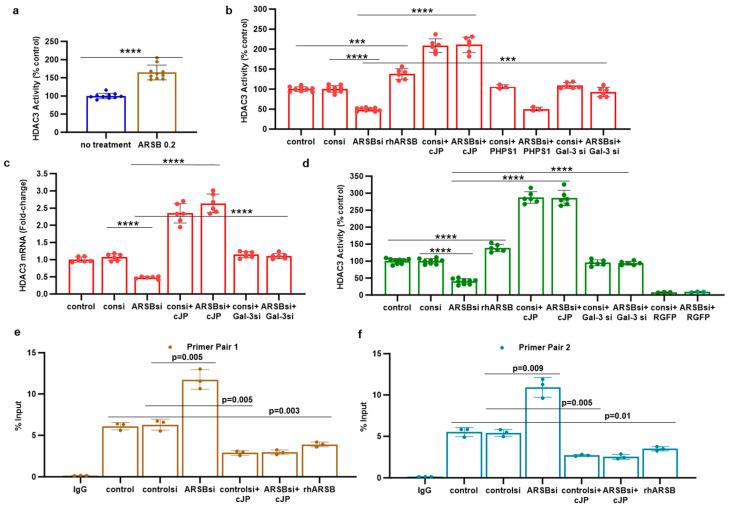
HDAC3 is increased, and promoter H3-acetylation is reduced by exogenous ARSB. (**a**) In the B16F10 tumors, histone deacetylase (HDAC)3 activity was increased by over 50% following local treatment by exogenous ARSB (0.2 mg/kg × 3 doses) (*p* < 0.001, n = 9). (**b**) In the A375 melanoma cells, ARSB silencing reduced (*p* < 0.0001, n = 9) and rhARSB increased (*p* < 0.001, n = 6) the HDAC3 activity. Treatment by c-Jun mimetic peptide (cJP) increased the HDAC3 activity to 200% of the control level. The SHP2 inhibitor PHPS1 did not affect the ARSB siRNA-induced decline, but galectin-3 silencing restored the HDAC-3 activity to near baseline (*p* < 0.001, n = 6). (**c**) HDAC3 expression was reduced by ARSB siRNA (*p* < 0.0001, n = 6) and normalized by galectin-3 silencing (*p* < 0.0001, n = 6). HDAC3 mRNA was increased to 2.3 ± 0.3 times the baseline following treatment by cJP. (**d**) In the normal human melanocytes, similar effects of ARSB siRNA, cJP, and galectin-3 siRNA were apparent as in the malignant A375 cells (*p* < 0.0001). RGFP699 completely blocked the HDAC3 activity. (**e**,**f**) A375 melanoma cells, including cells treated by ARSB siRNA, control siRNA, control si + cJP, ARSB siRNA + cJP, and rhARSB (1 ng/mL × 24 h), were fixed with 1% formaldehyde, chromatin was sheared, and soluble chromatin fragments were incubated with acetyl-H3 antibodies. Protein–DNA complexes were precipitated, DNA was immunoprecipitated by the reversal of cross-linking, and % Input DNA was measured using two sets of primers upstream of the PD-L1 start codon. Treatment of the cells by ARSB siRNA was associated with increased % Input DNA, in contrast to declines following treatment with cJP or rhARSB. *p*-values were determined by unpaired *t*-test, two-tailed, with unequal variance, and n ≥ 6. [ARSB = arylsulfatase B = N-acetylgalactosamine-4-sulfatase; consi = control siRNA; gal-3 = galectin-3; cJP = c-Jun mimetic peptide; RGFP = RGFP966 = HDAC3 inhibitor; rh = recombinant human; si = small interfering siRNA; 0.2 = 0.2 mg/kg treatment dose]. **** represents *p* ≤ 0.0001 and *** represents *p* ≤ 0.001.

Reports of mechanisms linking c-Jun and HDAC3 in the regulation of transcription [22,34,35] encouraged experiments to address the impact of exogenous ARSB and ARSB silencing on HDAC3 expression and activity. In the subcutaneous mouse melanoma tissue, HDAC3 activity increased following exogenous ARSB (*p* < 0.0001) (Figure 4a), in contrast to the decline in free galectin-3 (Figure 2a). In the A375 and M0 cells, HDAC3 activity decreased when ARSB was silenced and increased following exogenous ARSB (Figure 4b,d). The mRNA expression of HDAC3 decreased in the A375 cells when ARSB was silenced (Figure 4c). The inhibitory c-Jun mimetic peptide cJP increased the baseline HDAC3 activity and expression (Figure 4b–d). Galectin-3 silencing partially reversed the ARSB silencing-associated decline in HDAC3 (Figure 4b–d). In contrast, PHPS1 had no effect. The HDAC3 inhibitor RGFP966 completely blocked the HDAC3 activity in the M0 cells.

### 2.5. Regulation of PD-L1 Promoter by H3 Acetylation

To determine if the effects on HDAC3 affected the H3 acetylation of the PD-L1 promoter, the promoter was probed for H3 acetylation by a ChIP assay using two sets of promoter primers. A375 cells were treated by ARSB siRNA, control siRNA, ARSB siRNA + cJP, control siRNA + cJP, and rhARSB, as well as untreated control. DNA oligonucleotides were precipitated by acetyl-H3 antibody and control IgG antibody. The % DNA input was determined by QPCR and showed significant increases following ARSB silencing (Figure 4e,f). In contrast, rhARSB and treatment by cJP reduced the % DNA input from the control and control siRNA values. These results suggest that open chromatin followed ARSB knockdown and the associated decline in HDAC3 and increase in H3 acetylation, leading to an enhanced transcription of PD-L1.

### 2.6. Impact of β-D-Xyloside on Mediators of PD-L1 Expression

**Figure 5:**
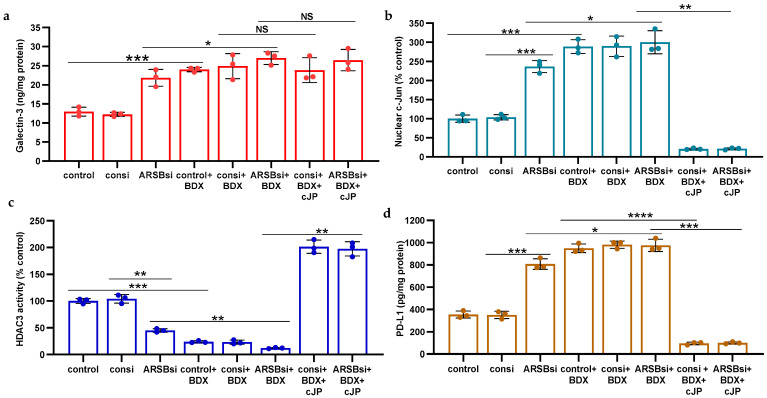
Impact of methyl-β-d-xylopyranoside (BDX) on mediators of PD-L1 expression. (**a**) Methyl-β-d-xylopyranoside (BDX), an inhibitor of chondroitin sulfate proteoglycan biosynthesis, increases free galectin-3 in the A375 cells (*p* ≤ 0.001, n = 3). The combination of ARSB siRNA and BDX has a slightly greater effect than ARSB siRNA alone (*p* < 0.05, n = 3). (**b**) Nuclear c-Jun is increased by BDX (*p* ≤ 0.001, n = 3) and ARSB siRNA (*p* ≤ 0.001, n = 3). cJP inhibits the effects of BDX and ARSB siRNA. (**c**) HDAC3 activity is reduced by both ARSB siRNA (*p* = 0.002, n = 3) and BDX (*p* = 0.0002, n = 3) and further reduced by their combination (*p* = 0.002, *p* = 0.003). These effects are reversed by cJP. (**d**) PD-L1 expression is increased by both ARSB siRNA and BDX, and these increases are inhibited by cJP. *p*-values are determined by unpaired *t*-test, two-tailed, with unequal variance. [ARSB = arylsulfatase B = N-acetylgalactosamine-4-sulfatase; BDX = methyl-β-d-xylopyranoside; consi = control siRNA; cJP = c-Jun mimetic peptide; HDAC = histone deacetylase; NS = not significant; PD-L1 = programmed death ligand-1; si = small interfering siRNA; 0.2 = 0.2 mg/kg treatment dose]. **** represents *p* ≤ 0.0001, *** represents *p* ≤ 0.001, ** represents *p* ≤ 0.01, and * is for *p* ≤ 0.05.

The impact of treatment by methyl-β-d-xylopyranoside (BDX) on free galectin-3 (Figure 5a), nuclear c-Jun (Figure 5b), HDAC3 activity (Figure 5c), and PD-L1 (Figure 5d) was examined in the A375 cells. A375 cells were exposed to BDX (1 mM × 48 h), in conjunction with ARSB or control silencing and with or without cJP (400 µM). BDX acts to inhibit the construction of functional proteoglycans by impairing the attachment of chondroitin sulfate chains to the core protein of proteoglycans [36]. The experiments indicate increases in galectin-3, nuclear c-Jun, and PD-L1 and a decline in HDAC3 activity by BDX. The combined effect of ARSB siRNA with BDX is greater than the independent effect of ARSB silencing. 

### 2.7. Overall Pathway of PD-L1 Expression by ARSB

**Figure 6:**
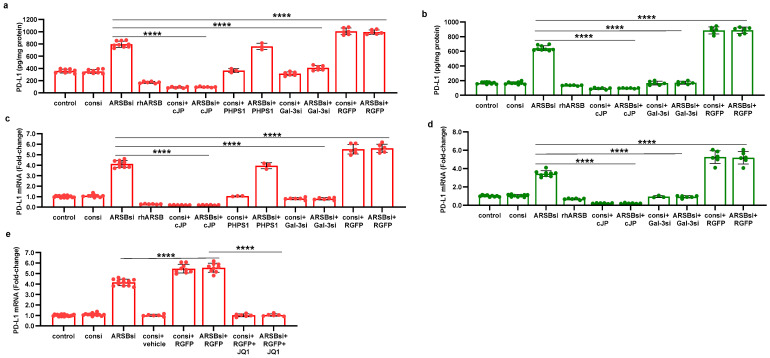
Effects of inhibition of c-Jun, galectin-3, and HDAC3 on ARSB-mediated changes in PD-L1 expression in A375 melanoma cells and normal melanocytes. (**a**) In the A375 cells, PD-L1 protein is markedly increased by ARSB siRNA, and this increase is inhibited by cJP and galectin-3 siRNA. PHPS1 does not affect the PD-L1 expression. The HDAC3 inhibitor, RGFP966, further increases the ARSB siRNA-induced increase in PD-L1. (**b**) In normal melanocytes, similar effects of ARSB siRNA, cJP, galectin-3 siRNA, and RGFP are observed. The total production of PD-L1 is less than in the A375 cells (642 ± 34 pg/mg protein vs. 796 ± 50 pg/mg protein; *p* < 0.0001, n = 9). (**c**,**d**) Similar effects are observed in PD-L1 mRNA expression in the A375 malignant cells and the normal melanocytes (*p* < 0.0001). Galectin-3 siRNA inhibits the effect of ARSB silencing, consistent with dependence on galectin-3 for the observed effects. (**e**) JQ1, the inhibitor of the BET family of bromodomain proteins, blocks the effects of ARSB siRNA and RGFP on PD-L1 expression (*p* < 0.0001). *p*-values are determined by an unpaired *t*-test, two-tailed, with unequal variance. [ARSB = arylsulfatase B = N-acetylgalactosamine-4-sulfatase; consi = control siRNA; gal-3 = galectin-3; cJP = c-Jun mimetic peptide; JQ1 = inhibitor of the BET family of bromodomain proteins; PD-L1 = programmed death ligand-1; PHPS1 = SHP2 inhibitor; RGFP = RGFP966 = HDAC3 inhibitor; rh = recombinant human; si = small interfering siRNA; 0.2 = 0.2 mg/kg treatment dose]. **** represents *p* ≤ 0.0001.

**Figure 7:**
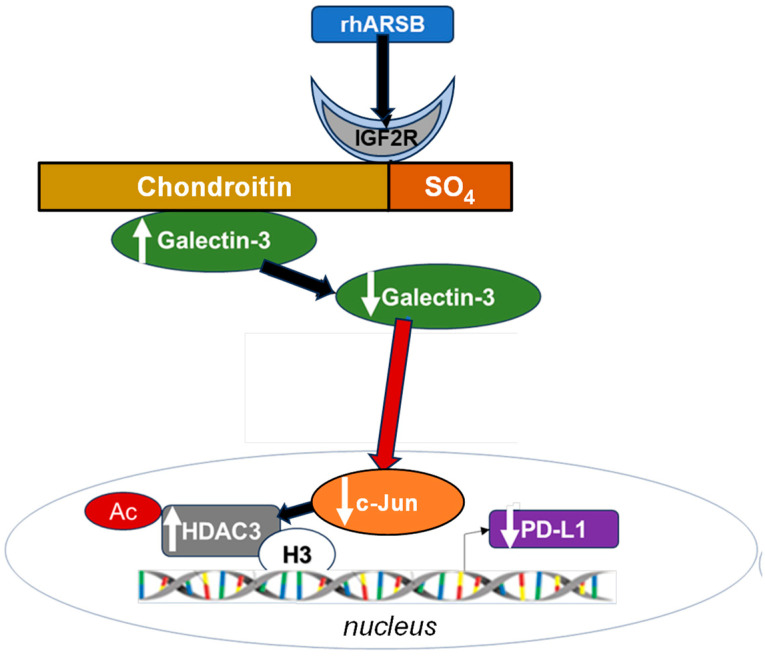
Schematic of overall pathway. Following exposure to bioactive, recombinant human ARSB, the sulfate group of chondroitin 4-sulfate is removed and galectin-3 binding with C4S is enhanced. This leads to a decline in the free galectin-3 and reduced impact on c-Jun DNA binding. Reciprocal effects lead to an increase in HDAC3, reduced acetyl-H3, increased availability of histone lysines to bind with PD-L1 promoter DNA, leading to closed chromatin, and reduced expression of PD-L1. When ARSB is silenced, opposite effects occur due to the increased availability of galectin-3.

The pathway by which exogenous ARSB decreases and knockdown of ARSB increases PD-L1 expression involves galectin-3, nuclear c-Jun, HDAC3, and acetyl-H3, and it leads to effects on the activation of the PD-L1 promoter. The impact of ARSB siRNA, exogenous ARSB, and inhibitors (including galectin-3 siRNA, cJP, JIP1, RGFP966, and JQ1) on PD-L1 expression in A375 and M0 cells is presented (Figure 6a–e) and summarized schematically (Figure 7).

## 3. Discussion

This report indicates that exogenous, bioactive ARSB reduces PD-L1 expression in B16F10 subcutaneous murine melanomas and in normal human melanocytes and A375 melanoma cells. Inversely, silencing ARSB increases PD-L1 expression in the cell lines. The impact on expression occurs due to an epigenetic mechanism involving HDAC3 and H3 acetylation. Initial measurements of PD-L1 in human prostate cell lines, including PC-3 cells, normal prostate epithelial cells, and normal prostate stromal cells, showed that PD-L1 mRNA expression increased to 1.8 times, 2.2 times, and 1.6 times the baseline level when ARSB was silenced (Supplementary Figure S2a). Other measurements from a dietary study of prediabetes showed that the expression of PD-L1 in circulating mononuclear cells declined by 62% when mononuclear ARSB activity increased by 34% (Supplementary Figure S2b) [37].

PD-L1 has emerged as a major focus in the immune-mediated control of tumor proliferation. The PD-1–PD-L1 binding between tumor-invading immune cells and tumor cells has been elucidated as a checkpoint which impedes other, potentially more cytotoxic, immune cell–tumor cell interactions. Many excellent clinical responses arise from treatment by checkpoint inhibitors using antibodies directed at either PD-1 or PD-L1; however, responses may not endure, some patients do not respond, and treatment toxicity may be severe [1,2,3,4,5]. Recognition that exogenous ARSB reduces PD-L1 expression may help to focus interest on chondroitin sulfate and on chondroitin sulfatases in cancer biology and in the regulation of immune responses.

The production of PD-L1 in the murine subcutaneous B16F10 melanomas and in the cultured A375 melanoma cells is one of many products of these malignant cells. Many other melanoma small-molecule products, including cytokines, hormones, and small signaling molecules, are implicated in potential system-wide interactions [38]. The pathway of melanin production is inherent in these cells and may impact proliferation and cell fate. The production of melanin and intermediates, including quinones, semi-quinones, and reactive oxygen species, can impact melanoma progression and responsiveness to therapy [39]. Intersections between melanogenesis and sulfate/sulfatase biology are not clarified, but previous work showed that a decline in ARSB was associated with increased phosphorylation and transcriptional effects of MITF (microphthalmia-associated transcription factor; melanocyte-inducing transcription factor) in hepatic cells [40]. Since MITF controls the expression of genes required for melanin synthesis, including tyrosinase [41], future elucidation of the specific effects between sulfation and melanogenesis may provide new insight into the complex biology underlying melanogenesis and melanoma.

Recent work demonstrated that exogenous, bioactive ARSB reduced the progression and improved the survival of mice with B16F10 subcutaneous melanomas [6], and further evaluation of the potential therapeutic benefit of exogenous ARSB treatment is warranted. The treatment benefit of rhARSB occurred at a dose of 0.2 mg/kg SQ on days 2, 7, and 14 post tumor inoculation, with no evidence of toxicity. Recombinant human ARSB is used successfully in enzyme replacement therapy of congenital ARSB deficiency, Mucopolysaccharidosis VI, by the administration of a weekly dose of 1.0 mg/kg IV [42].

In cultured prostate stem and epithelial cells, the effects of ARSB on signaling events, including increase in phospho-JNK, were attributed to the enhanced binding of SHP2 with C4S when ARSB activity was reduced, leading to sustained phosphorylation of vital signaling molecules [8,9,11]. In the current experiments, the SHP2 inhibitor PHPS1 had no impact on PD-L1 expression and did not affect nuclear c-Jun, phospho-JNK, or HDAC3 activity. The mechanism by which phospho-JNK is modified in the melanoma cells appears to be independent of SHP2 and is not yet clarified. The epigenetic mechanism in the melanoma cells affecting PD-L1 expression resembles the previously observed mechanisms by which ARSB and chondroitin 4-sulfation affect galectin-3 and AP-1, as shown by transcriptional effects on HIF-1α, Wnt9a, and versican [10,12,13]. The impact of ARSB on chondroitin 4-sulfation and, thereby, on the binding of galectin-3 and restriction of free galectin-3, causes exogenous ARSB to act as an inhibitor of galectin-3. The inhibition of galectin-3 is under intense investigation as a cancer treatment approach, including immunosuppression and interference with PD-1-PD-L1 interaction in melanoma and other malignancies [43,44,45,46,47]. 

The treatment of the melanoma cells by methyl-β-d-xylopyranoside (BDX) produced similar effects as ARSB knockdown. The effects included an increase in free galectin-3, an increase in nuclear c-Jun, a decline in HDAC3 activity, and an increase in PD-L1 expression. Together, ARSB silencing and BDX further increased the effect of ARSB siRNA. The xylosides are reported to initiate non-proteoglycan-based glycosaminoglycan (GAG) chain elongation, thereby competing with endogenous proteoglycan synthesis [36,48,49,50,51,52,53]. They can compete for critical enzymes, such as β-1,4-galactosyltransferase 7 (β4GalT7), which are required for normal proteoglycan synthesis [50]. Detailed investigations are ongoing to consider how variously modified, synthetic xylosides act as mimetics of vital, endogenous carbohydrates and how they interact with lectins [51,52,53]. An investigation of the interactions between xylosides and sulfatases, galactosyltransferases, and sulfotransferases may help to elucidate fundamental biological mechanisms. An identification of the impact of ARSB-induced changes in chondroitin 4-sulfate on free galectin-3, c-Jun, HDAC3, and histone acetylation, as presented in this report, may help to explain how sulfation contributes to the regulation of transcriptional events and to malignant progression.

## 4. Materials and Methods

### 4.1. Cell Culture

A375 human melanoma cells (CRL-1619, ATCC, Manassas, VA, USA) were grown in Dulbecco’s modified Eagle medium (DMEM) supplemented with 10% fetal bovine serum (FBS; ATCC), and 1% penicillin–streptomycin (ATCC). The cells were maintained at 37 °C in a humidified, 5% CO_2_ environment with media exchange every 2 days. Confluent cells in T-25 flasks were harvested by EDTA-trypsin (ATCC) and sub-cultured. Primary normal melanocytes were cultured in Airway Epithelial Cell Basal Medium (ATCC, Manassas, VA, USA) with a melanocyte growth kit (ATCC). The cells were maintained at 37 °C in a humidified, 5% CO_2_ environment with media exchange every 3 days. Confluent cells in T-25 flasks were harvested by trypsin for primary cells (ATCC) and sub-cultured. B16F10 mouse melanoma cells were purchased (ATCC CRL-6475), and these melanin-producing cells were cultured in DMEM supplemented with 10% FBS and 1% penicillin–streptomycin. Cells were screened for pathogens by IDEXX BioAnalytics (Columbia, MO, USA). Cells were maintained at 37 °C in a humidified, 5% CO_2_ environment with media exchange every 2 days, and confluent cells in T-25 flasks were harvested by EDTA-trypsin and sub-cultured.

### 4.2. Animal Procedures

Eight-week-old female C57BL/6J mice (n = 40) were purchased (Jackson Laboratories, Bar Harbor, ME, USA) and housed in the Veterinary Medicine Unit at the Jesse Brown VA Medical Center (JBVAMC, Chicago, IL, USA) [6]. Principles of laboratory animal care were followed, and all procedures were approved by the AALAC accredited Animal Care Committee of the JBVAMC. Mice were fed a standard diet and maintained in groups of three in a cage with routine light–dark cycles. Mice were inoculated subcutaneously with 2.5 × 10^5^ B16F10 mouse melanoma cells (ATCC) in 100 μL of normal saline. Treatment by injection of recombinant, human, bioactive ARSB, which was expressed in *E. coli* (R&D Systems, Minneapolis, MN, USA), or normal saline control injection was started 48 h following tumor inoculation. Recombinant ARSB was diluted in sterile, normal saline and injected subcutaneously around the tumor with a 25-gauge needle at a dose of a 0.2 mg/kg body weight on days 2, 7, and 14 [6]. Body weight and tumor volume were measured, and the volume was expressed in cm^3^ [0.5 × L × W^2^] (L = long diameter; W = short diameter of the tumor).

### 4.3. Treatment of A375 Human Melanoma Cells by Exogenous ARSB, siRNAs, and Other Agents

A375 cells were treated by exogenous, bioactive rhARSB (1 ng/mL × 24 h; R&D, Biotechne, Minneapolis, MN, USA). ARSB, galectin-3, mannose-6 phosphate receptor, and insulin-like growth factor 2 receptor were silenced by specific siRNA in the A375 cells at 70% confluence using standard procedures and verified siRNA (Invitrogen, Thermo Fisher Scientific, Waltham, MA, USA). Media were exchanged after 24 h, and cell treatments were initiated. Treatments were performed for 24 h, unless indicated otherwise, and included the following:

cJP (400 μM with 2 h pre-incubation and a total of 26 h of exposure; #1989, R&D Systems, Bio-Techne, Minneapolis, MN, USA), a cell-permeable c-Jun mimetic peptide [54]; JIP-1 (10 μM with 2 h pre-incubation and a total of 26 h of exposure; #1565, Tocris, Bio-Techne), an inhibitor of activated JNK based on interacting protein-1 [55]; RGFP966 (5 µM, SelleckChem, Houston, TX, USA), an HDAC3 inhibitor; PHPS1 (phenylhydrazonopyrazolone sulfonate or PTPN11, 30 μM; Sigma-Aldrich, St. Louis, MO, USA), a chemical inhibitor of SHP2, the tyrosine–protein phosphatase non-receptor type 11; JQ1 (10 µM, MedChem Express, Monmouth Junction, NJ, USA), a thienotriazolodiazepine and a potent inhibitor of the BET family of bromodomain proteins [56]; and methyl β-d-xylopyranoside (BDX; 1 mM × 48 h, #M5878, Sigma-Aldrich). Some cell preparations were treated with BDX for 48 h in conjunction with ARSB or control siRNA and with or without cJP (400 µM) for the 24–48 h time period.

### 4.4. Treated and Control Cells Were Harvested and Frozen at −80 °C for Subsequent Analysis

Small interfering (si) RNAs for ARSB (Qiagen), galectin-3 (Qiagen), mannose-6-phosphate receptor (#s8375; Thermo Fisher, Waltham, MA, USA), and insulin-like growth factor 2 receptor (IGF2R) (#s7218, Thermo Fisher) were validated by QRT-PCR to confirm effective inhibition. The siRNA sequences for ARSB (NM_000046) silencing were as follows: sense 5′-GGGUAUGGUCUCUAGGCA-3′ and antisense: 5′-UUGCCUAGAGACCAUACCC-3′. The sequence of the DNA template for human galectin-3 silencing (Hs_ LGALS3_9) was 5′-ATGATGTTGCCTTCCACTTTA-3′. A375 cells and normal melanocytes were grown to ~60% confluence and then silenced by adding 0.6 μL of 20 μM siRNA (150 ng) mixed with 100 μL of a serum-free medium and 12 μL of HiPerfect Transfection Reagent (Qiagen, Germantown, MD, USA).

Human ARSB (NM_000046) plasmid in a pCMV6-Entry vector was obtained (#RC214604; Origene, Germantown, MD, USA), and A375 cells were transfected with 2 µg of the plasmid or empty vector using Lipofectamine^TM^ 2000 transfection reagent (Thermo Fisher) and Opti-MEM^TM^ reduced serum medium (Thermo Fisher). Media were exchanged after 6 h, and cells were incubated under standard conditions and harvested 24 h after transfection. 

### 4.5. Immunohistochemistry of PD-L1 in A375 Cells

A-375 human melanoma cells were plated in compartment slides and grown to 70–80% confluence. Cells were treated with ARSB siRNA (Qiagen) or control siRNA (Qiagen) for 24 h or by recombinant human ARSB (1 ng/mL × 24 h). Preparations were then fixed for 2 h with 2% paraformaldehyde and washed in 1×-PBS containing 1 mM calcium chloride (pH 7.4) for 5 min. Cells were then incubated with 5% normal goat serum (#501972, Invitrogen, Thermo Fisher) for blocking and permeabilized with 0.08% saponin in 1×-PBS/Ca^++^/saponin. Slides were incubated overnight with PD-L1 antibody (#13684, Cell Signaling, Danvers, MA, USA) at 4 °C, washed four times in 1×-PBS/Ca^++^/saponin, and then stained with secondary antibody rabbit anti-mouse FITC IgG (1:100, ab8517, Abcam, Waltham, MA, USA) for 1 h. Slides were washed four times in 1×-PBS/Ca^++^/saponin and coverslipped using ProLong™ Gold anti-fade reagent with DAPI (P36941, Invitrogen) for nuclear staining. The fluorochromes were scanned using EVOS M5000 Imaging System (Thermo Fisher) at 20×. The captured TIFF images were exported for analysis and reproduction.

### 4.6. Arylsulfatase B (ARSB) Activity Assay

ARSB measurements were performed using a fluorometric assay, following a standard protocol with 20 μL of cell homogenate prepared in ddH_2_O and 80 μL of an assay buffer (0.05 M Na acetate buffer with 20 mM barium sulfate pH 5.6 at 37 °C) with 100 μL of substrate (5 mM 4-methylumbelliferyl sulfate in fresh assay buffer) in a black microplate, as previously reported [8].

### 4.7. Measurement of Total Sulfated Glycosaminoglycans (sGAGs) and Chondroitin-4-Sulfate

Total sulfated glycosaminoglycans (sGAGs) were measured in the cell extracts by a sulfated GAG assay (Blyscan^™^, Biocolor Ltd., Newtownabbey, Northern Ireland, UK), as previously described [10]. Chondroitin sulfate (CS) or chondroitin 4-sulfate (C4S) in the samples was determined following immunoprecipitation by dynabeads (Life Technologies, Carlsbad, CA, USA) coated with a total CS antibody (CS-56, Abcam, Waltham, MA, USA) or C4S antibody (LY111, Amsbio, Cambridge, MA, USA). Beads were mixed with samples and incubated, and the immunoprecipitated CS molecules were eluted and subjected to the Blyscan sulfated GAG assay, as above.

### 4.8. Total Sulfotransferase Activity

Total Sulfotransferase activity was determined using the Universal Sulfotransferase Activity kit (R&D Systems, Minneapolis, MN, USA). The activity results were normalized using the total cellular protein and expressed as a percentage of the control value.

### 4.9. ELISAs for Galectin-3, Phospho-(Thr183/Tyr185)-JNK, PD-L1

Galectin-3 in the cell lysates was determined by a sandwich ELISA kit (R&D Systems) for human galectin-3. The wells of a microtiter plate were coated with a specific anti-galectin-3 monoclonal antibody, and nonspecific sites were blocked by a blocking buffer with 1% bovine serum albumin (BSA). Sample galectin-3 captured in the microtiter wells was detected by biotin-conjugated secondary galectin-3 antibody and streptavidin-horseradish peroxidase (HRP). Hydrogen peroxide-tetramethylbenzidine (TMB) chromogenic substrate was used to develop the color, and the color intensity was measured at 450 nm in an ELISA plate reader (FLUOstar, BMG Labtech, Inc., Cary, NC, USA). The galectin-3 concentrations were extrapolated from a standard curve, and sample values were normalized using total protein content. Galectin-3 in mouse tumor tissues was determined by a similar mouse sandwich ELISA kit (R&D Systems). Galectin-3 was also measured following the immunoprecipitation of mouse tumor treated and control tissue lysates with the C4S antibody. Chondroitin 4-sulfate was immunoprecipitated from the cell lysates, as previously described, and the immunoprecipitate was eluted with a dye-free elution buffer and subjected to mouse galectin-3 ELISA.

Cell extracts were prepared from both treated and control cells in a cell lysis buffer (Cell Signaling Technology, Danvers, MA, USA; 9803S). Tumor tissues from treated and untreated animals were homogenized for the measurement of phospho-(Thr183/Tyr185)-JNK (c-Jun N-terminal kinase 1). Phospho-JNK was measured in cell and tissue samples using a DuoSet sandwich ELISA kit (R&D Systems). Samples and standards were added to the wells of the microtiter plate precoated with a capture antibody to human JNK. Phospho-JNK in the lysates was captured by the coated antibody on the plate and detected with a biotinylated antibody to phospho-JNK. Streptavidin-HRP and hydrogen peroxide/TMB substrate were used to develop color, which was read at 450 nm in a plate reader (FLUOstar). Phospho-JNK concentrations in the samples were extrapolated from a curve derived using known standards and expressed as % control.

Cell extracts were prepared from both treated and untreated control cells in a cell lysis buffer. Malignant tumor issues from treated and untreated mice were homogenized. PD-L1 was measured in cell and tissue samples using a DuoSet sandwich ELISA kit (R&D Systems). Samples and standards were added to the wells of the microtiter plate precoated with a capture antibody to human/mouse PD-L1. PD-L1 in the lysates was captured by the coated antibody on the plate and detected with a biotinylated antibody to PD-L1. Streptavidin-HRP and hydrogen peroxide/TMB substrate were used to develop color proportional to the bound HRP activity. The reaction was stopped, and the optical density of the color was read at 450 nm in a plate reader (FLUOstar). PD-L1 concentrations in the samples were extrapolated from a curve derived using known standards. PD-L1 was also measured by ELISA (Abcam, Waltham, MA, USA; ab278124) following immunoprecipitation with a chondroitin sulfate antibody (CS-56, Abcam; ab11570), which reacts with C4S and chondroitin 6-sulfate.

### 4.10. Oligonucleotide-Based ELISA to Detect Nuclear c-Jun

Oligonucleotide binding assay (TransAM Kit, Active Motif, Carlsbad, CA, USA) was used to detect nuclear c-Jun in the rhARSB-treated and control mouse melanoma tissue, in A375 melanoma cells, and in normal melanocyte cells. Nuclear extracts were prepared using a nuclear extract preparation kit (Active Motif, Carlsbad, CA, USA) and were added to the wells of a 96-well microtiter plate pre-coated with the AP-1 consensus oligonucleotide sequence (5′-TGAGTCA-3′). The bound c-Jun was recognized by a specific c-Jun primary antibody and finally detected by an HRP-conjugated secondary antibody. Color was developed using hydrogen peroxide/TMB substrate and was proportional to the activity of the bound HRP. The reaction was stopped by an acid stop solution, and the optical density of the color was read at 450 nm in a plate reader (FLUOstar). Sample values were normalized by total cell protein and expressed as a percentage of the untreated control.

### 4.11. HDAC3 Activity and Expression

Histone Deacetylase (HDAC)3 in the tissue and cell samples was determined following immunoprecipitation with a specific HDAC3 antibody (Rabbit mAb #85057, Cell Signaling Technology, Inc., Danvers, MA, USA). Dynabeads (Life Technologies, Carlsbad, CA, USA) were coated with a specific HDAC3 (D201K) antibody, and beads were mixed with samples, incubated, and immunoprecipitated. Immunoprecipitated HDAC3 molecules were eluted and subjected to an HDAC Activity Assay (ab156064, Abcam, Cambridge, MA, USA). An assay buffer and a substrate peptide were added to the wells of a black microtiter plate. Reactions were initiated by adding 5 μL of the buffer for the no-enzyme control assay or the enzyme sample to each well, mixed thoroughly, and incubated for 20 min at room temperature (RT). Then, 20 μL of a stop solution was added to each well of the microtiter plate and mixed thoroughly, before adding 5 μL of a developer to each well and mixing thoroughly. Following incubation for 20 min at RT, fluorescence intensity was read at Ex/Em = 350–380 nm/440–460 nm. HDAC3 activity was expressed as % control.

### 4.12. mRNA Expression of HDAC3 and PDL1

Total RNA was prepared from treated and control cells using an RNeasy Mini Kit (Qiagen, Germantown, MD, USA). Equal amounts of purified RNAs from the control and treated cells were reverse-transcribed and amplified using a Brilliant SYBR Green QRT-PCR Master Mix (BIO-RAD, Hercules, CA, USA). Human β-actin was used as an internal control. QRT-PCR was performed using the following specific primers:

PDL1 human (NM_014143) forward: 5′-TTTACTGTCACGGTTCCCAAG-3′ and reverse: 5′-GCTGAACCTTCAGGTCTTCCT-3′;

PDL1 mouse (NM_021893) forward: 5′-AGTTTGTGGCAGGAGAGGAG-3′ and reverse: 5′-CTGGTTGATTTTGCGGTATG-3′.

HDAC3 human (NM_003880) forward: 5′-GAGTTCTGCTCGCGTTACACAG-3′ and reverse: 5′-CGTTGACATAGCAGACAGAG-3′;

HDAC3 mouse (NM_010411) forward: 5′-AACCTCATCGCCTGGCATTGAC-3′ and reverse: 5′-GTAGTCCTCAGAGAAGCGG-3′.

### 4.13. Chromatin Immunoprecipitation (ChIP) Assay for Histone 3-Acetylation

Chromatin immunoprecipitation assays were performed using the Acetyl-Histone H3 Immunoprecipitation (ChIP) Assay Kit (#17–245, MilliporeSigma, Burlington, MA, USA). A375 melanoma cells were fixed with 1% formaldehyde for 10 min at room temperature. This was followed by the shearing of chromatin by sonication on ice to obtain DNA lengths between 200 and 1000 base pairs. Soluble chromatin fragments of 200 to 1000 bp in length were incubated with 5 μg of acetyl-histone H3 (Lys9) rabbit monoclonal antibody (C5B11; #9649, Cell Signaling) at 4 °C overnight. Rabbit IgG was used as a negative control for validating the ChIP assay. Protein–DNA complexes were precipitated by protein A/G-coupled magnetic beads. DNA was purified from the immunoprecipitated complexes by a reversal of cross-linking and followed by proteinase K treatment. Real-time RT-PCR was performed using an SYBR Green QRT-PCR master mix (Bio-Rad, Des Plaines, IL, USA). Two sets of ChIP primers covering 1800 bp upstream of the human *PD-L1* gene start codon and designed by NCBI-Blast software (Prizm 10-1-2; Microsoft 265) were as follows: primer set 1 (−1178 bp to −1117 bp), forward 5′-GCT GGG CCC AAA CCC TAT T-3′ and reverse 5′-TTT GGC AGG AGC ATG GAG TT-3′; primer set 2 (−455 bp to −356 bp), forward 5′-ATG GGT CTG CTG CTG ACT TT-3′ and reverse 5′-GGC GTC CCC CTT TCTGAT AA-3′ [25]. The ChIP qPCR result was calculated using the ΔΔCt method. Each ChIP fractions’ Ct value was normalized to the input DNA fraction and expressed as % Input.

### 4.14. Statistical Analysis

Data presented are the mean ± SD of at least six independent experiments, unless stated otherwise. Statistical significance was determined by unpaired *t*-tests, two-tailed, corrected for unequal variance using Microsoft 365 Excel or Prizm 10.1.2 software (GraphPad, La Jolla, CA, USA), unless stated otherwise. In the figures, **** represents *p* ≤ 0.0001, *** represents *p* ≤ 0.001, ** represents *p* ≤ 0.01, and * is for *p* ≤ 0.05. In the graphs, the top of the bar is the mean value and horizontal bars indicate one standard deviation. Each dot represents an independent experiment.

## 5. Conclusions

This is the first demonstration of epigenetic effects of exogenous, bioactive ARSB. The recombinant, human ARSB was previously shown to reduce tumor volume and improve the survival of mice with subcutaneous B16F10 melanomas. The treatment of melanoma cells and B16F10 melanomas by rhARSB leads to increased HDAC3, reduced acetyl-H3 antibody association with the PD-L1 promoter, and reduced PD-L1 expression. The ARSB-mediated pathway involves effects on chondroitin 4-sulfate, galectin-3, nuclear c-Jun, HDAC3, and H3 acetylation at the PD-L1 promoter. Effects of rhARSB were inhibited by insulin-like growth factor-2 receptor knockdown. Both ARSB siRNA and methyl β-d-xylopyranoside increased free galectin-3, and rhARSB reduced free galectin-3. The findings indicate that rhARSB may be an effective treatment for malignant melanoma and may influence the outcome of checkpoint inhibitor therapy.

## Data Availability

Data are available by communication with J.K.T.

## Supplementary Materials

The supporting information can be downloaded at: https://www.mdpi.com/article/10.3390/ijms25115851/s1.
