# Supplementary material for: N-Acetylgalactosamine-4-sulfatase (Arylsulfatase B) Regulates PD-L1 Expression in Melanoma by an HDAC3-Mediated Epigenetic Mechanism
Source: Int J Mol Sci. 2024 May 28;25(11):5851. doi: 10.3390/ijms25115851 (PMC11172302; doi:10.3390/ijms25115851)
Supplement: Supplementary file 1 [file ijms-25-05851-s001.zip › ijms-2986934-supplementary.pdf]

Supplementary Figure S1. Effects of ARSB transfection on ARSB activity and PD-L1 mRNA expression

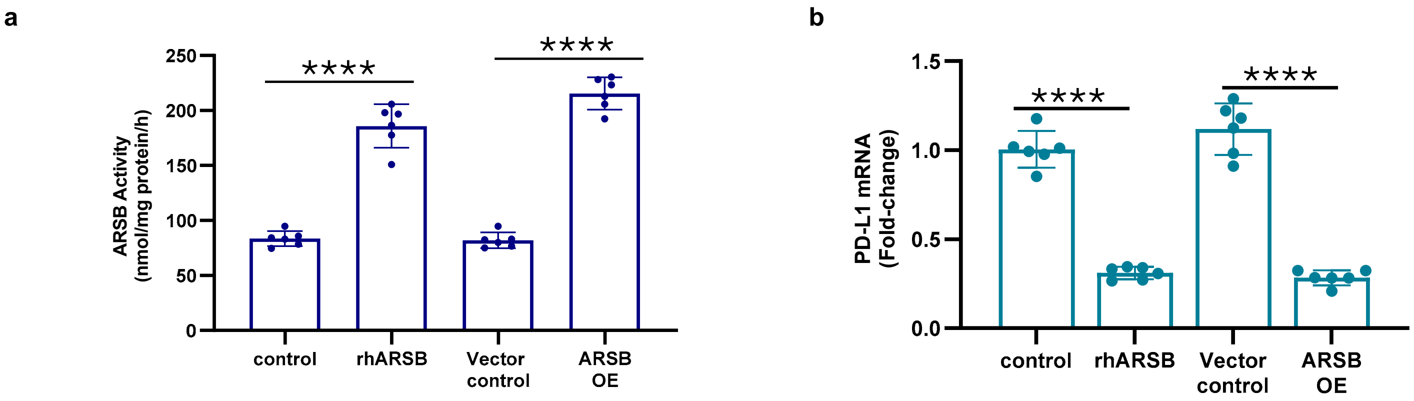

Supplementary Figure S1a. ARSB activity was measured using the substrate 4-methylumbelliferyl, as previously described (Ref.8), and expressed as nmol/mg protein/h, following treatment of A375 melanoma cells by rhARSB (1 ng/ml x 24h), empty vector control, or by ARSB plasmid. Activity significantly increased following treatment with rhARSB ($p<10^{-5}$; unpaired t-test, two-tailed, unequal variance).

S1b. PD-L1 expression was measured in the treated cells by QPCR and declined by 28% following rhARSB and by 31% following ARSB transfection. Declines in PD-L1 mRNA are highy signficant (**** $p<10^{-5}$; unpaired t-test,]two-tailed, unequal variance). [ARSB=arylsulfatase B; ND=no difference; OE=overexpression; rh=recombinant human].

Supplementary Figure S2. PD-L1 in other cells following changes in ARSB.

a. PD-L1 in prostate cells following ARSB knockdown

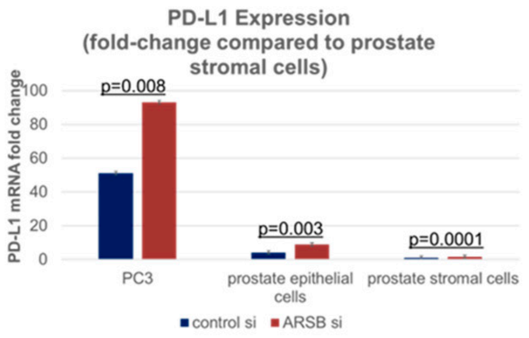

b. Human mononuclear cells PD-L1 declines following no-carrageenan diet

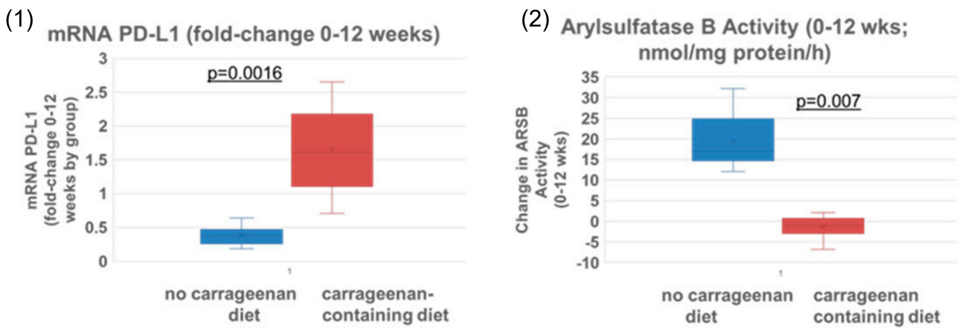

Supplementary Figure S2a. In human prostate cells lines (PC-3 (ATCC CRL-1435), prostate epithelial cells (ATCC CRL-2854) and prostate stromal cells (ATCC CRL-2850), PD-L1 mRNA increased significantly following treatment by ARSB siRNA.

S2b(1). In patients with pre-diabetes on a no-carrageenan diet for 12 weeks, mononuclear PD-L1 mRNA expression declined from baseline. In contrast, there was no decline in PD-L1 expression in cells of participants on a regular diet.

S2b(2). The decline in PD-L1 expression was associated with an increase in mononuclear ARSB activity in participants on the no-carrageenan diet.
